# Ultrashort Cationic Lipopeptides–Effect of *N*-Terminal Amino Acid and Fatty Acid Type on Antimicrobial Activity and Hemolysis

**DOI:** 10.3390/molecules25020257

**Published:** 2020-01-08

**Authors:** Damian Neubauer, Maciej Jaśkiewicz, Marta Bauer, Krzysztof Gołacki, Wojciech Kamysz

**Affiliations:** Department of Inorganic Chemistry, Faculty of Pharmacy, Medical University of Gdańsk, 80-416 Gdańsk, Poland; mj@gumed.edu.pl (M.J.); marta.bauer@gumed.edu.pl (M.B.); krzysztofgolackifarm@gumed.edu.pl (K.G.); wojciech.kamysz@gumed.edu.pl (W.K.)

**Keywords:** lipopeptides, cationic lipopeptides, USCLs, antimicrobial peptides, hydrophobicity, branched fatty acid

## Abstract

Ultrashort cationic lipopeptides (USCLs) are promising antimicrobial agents that hypothetically may be alternatively used to combat pathogens such as bacteria and fungi. In general, USCLs consist of fatty acid chains and a few basic amino acid residues. The main shortcoming of USCLs is their relatively high cytotoxicity and hemolytic activity. This study focuses on the impact of the hydrophobic fatty acid chain, on both antimicrobial and hemolytic activities. To learn more about this region, a series of USCLs with different straight-chain fatty acids (C8, C10, C12, C14) attached to the tripeptide with two arginine residues were synthesized. The amino acid at the *N*-terminal position was exchanged for proteinogenic and non-proteinogenic amino acid residues (24 in total). Moreover, the branched fatty acid residues were conjugated to *N*-terminus of a dipeptide with two arginine residues. All USCLs had C-terminal amides. USCLs were tested against reference bacterial strains (including ESKAPE group) and *Candida albicans*. The hemolytic potential was tested on human erythrocytes. Hydrophobicity of the compounds was evaluated by RP-HPLC. Shortening of the fatty acid chain and simultaneous addition of amino acid residue at *N*-terminus were expected to result in more selective and active compounds than those of the reference lipopeptides with similar lipophilicity. Hypothetically, this approach would also be beneficial to other antimicrobial peptides where *N*-lipidation strategy was used to improve their biological characteristics.

## 1. Introduction

**Figure 1:**
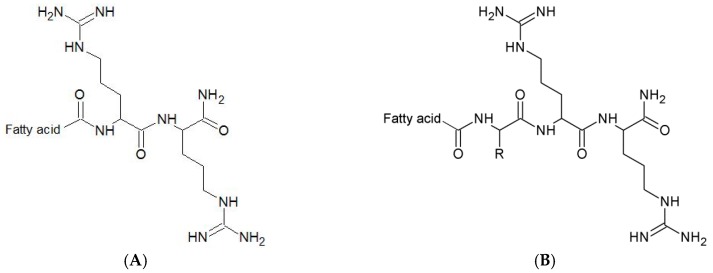
The general structure of the lipopeptides used: (**A**)—reference lipopeptides and lipopeptides with branched fatty acid chain; (**B**)—analogs with additional *N*-terminal amino acid (R). R–Side Chain of Substituted Residue.

Ultrashort cationic lipopeptides (USCLs) are a class of compounds that consist of a few amino acid residues (up to 7) and fatty acid chain(s). These molecules are amphiphilic owing to the presence of hydrophobic acid residue (tail) and hydrophilic peptide moiety (head). In general, lipopeptides have dispersing, foaming, de-emulsificating, and moisturizing properties [1]. Furthermore, it is worth nothing that USCLs offer promising antimicrobial activities against planktonic cells and biofilms of bacteria and fungi [2,3,4,5]. USCLs were also applied to modify materials to prevent bacterial colonization crucial to hard-to-treat biomaterial-associated infections [6]. Providing new effective agents to the treatment and prevention of infections is highly demanded, particularly in the light of escalating drug resistance. Antimicrobial peptides and lipopeptides are considered to be an alternative in the treatment of bacterial pathogens such as those constituting ESKAPE group (*Enterococcus faecium*, *Staphylococcus aureus*, *Klebsiella pneumoniae*, *Acinetobacter baumannii*, *Pseudomonas aeruginosa*, and *Enterobacter* spp.) which are recognized as being responsible for multi-drug resistance [7,8]. USCLs exhibit a detergent-like mode of action and hence their activity is based on disintegration of lipid bilayer. The main parameters considered to have substantial contribution to antimicrobial activity and toxicity of USCLs are the length of the fatty acid chain, net charge and the type of basic amino acids (usually arginine or lysine) [3,9,10]. According to the up-to-date epidemiological data resistance to commercially available lipopeptide drugs such as daptomycin, echinocandins, and polymyxins depends on a particular antibiotic, group of patients and geographical region; however it is still rare [11,12,13,14]. USCLs seem to be non-specific membrane disruptors and therefore the risk of development of resistance is relatively low especially as compared to that of available antibiotics [15]. Unfortunately, lipopeptides with high antimicrobial activity usually exhibit substantial toxicity against human cells including red blood cells (hRBCs; erythrocytes) [3,5,16]. The aim of this study was to analyze the hydrophobic region of the USCLs to find out how it could be modified to improve selectivity for pathogens over human cells. Compounds used in this study were derivatives of USCL with palmitic acid and two arginine residues and a C-terminal amide (C16-RR-NH_2_) that have proven antimicrobial activity [5,17,18]. Furthermore, arginine was chosen over lysine owing to its extensive H-bonding with phospholipid head-groups in the membrane and thus enhanced membrane perturbations [19]. The general structure of the lipopeptides used in this study is presented in Figure 1.

In this study the following straight chain fatty acids were used: C8, C10, C12, or C14, while as reference compounds, lipopeptides with two arginine residues only (Cx-RR-NH_2_; Cx–fatty acid residue, x = {8, 10, 12, 14, 16, 18}). It should be noted that using a longer fatty acid chain will result in enhanced lipophilicity of USCL. As a matter of fact, antimicrobial activity depends on hydrophobicity and therefore optimum hydrophobicity can be expected to result in the highest activity. In other words, both a higher or a lower lipophilicity of a compound would reduce its antimicrobial activity [16,20,21]. Moreover it is well documented that lipopeptides with C16 or C18 fatty acid chains are toxic to human cells, but it seems that there is some optimal chain length [16,21]. In this study, USCLs with different length of fatty acid chain and different amino acid residues at first position (*N*-terminus) were tested. Hypothetically, shortening of the fatty acid chain and the reduction in peptide hydrophobicity can be compensated by hydrophobic amino acid residues. The question that arises is how this fatty acid shortening and simultaneous addition of amino acids at *N*-terminus can affect in vitro biological activity? In order to respond to this question, the following proteinogenic and non-proteinogenic amino acids were used: Ala, Arg, Asn, Asp, Cys(Acm), Gln, Glu, Gly, His, Ile, Leu, Lys, Met, Met(O), Met(O2), norleucine (Nle), norvaline (Nva), Phe, Pro, Ser, Thr, Trp, Tyr, and Val. Unfortunately, lipopeptides with cysteine residues were unstable due to their high susceptibility to oxidation and therefore were not included in this study. The wide spectrum of the amino acids used provided a deep insight into the aspects other than hydrophobicity (net charge, hydrogen bonds, aromaticity). Fatty acids residues of natural antimicrobial lipopeptides are not only straight-chain compounds but can also be branched ones [22]. For instance, Polymyxin B is a natural antibiotic with a branched fatty acid chain linked to decapeptide with two major components, Polymyxin B_1_ and Polymyxin B_2_ containing 6-methyloctanoic and 6-methylheptanoic acids residues, respectively [23]. Moreover, to determine the impact of the branched structure of the fatty acid chain a series of lipopeptides with two arginine resides and *N*-terminal 2-ethylhexanoic, 2-butyloctanoic and 2-hexyldecanoic acid residues (C6(2), C8(4), C10(6)) were synthesized. To evaluate peptides hydrophobicity, RP-HPLC analyses were performed. Minimum Inhibitory Concentration of all lipopeptides were determined against reference strains of bacteria, namely *S. aureus* ATCC 25923, *P. aeruginosa* ATCC 9027 and the fungus *C. albicans* ATCC 10231. Moreover, for the most promising lipopeptides tests against ESKAPE strains, *E. faecium* ATCC 700221, *S. aureus* ATCC 33591, *K. pneumoniae* ATCC 700603, *A. baumannii* ATCC BAA-1605, *P. aeruginosa* ATCC 9027, *K. aerogenes* ATCC 13048 (previously known as *Enterobacter aerogenes*) were performed.

To sum up, position of additional hydrophobic amino acid in the peptide backbone was found to be crucial for relative lipopeptide hydrophobicity. In USCLs used in this study, *N*-terminal position with the hydrophobic amino acid (phenylalanine) resulted in the most lipophilic compound in the series and, while that with C-terminal phenylalanine was the least hydrophobic. Shortening of fatty acid chain and simultaneous addition of amino acid residues (usually hydrophobic) resulted in compounds with improved antimicrobial activity and selectivity. Moreover, the lipopeptide with a branched fatty acid chain, C10(6)-RR-NH_2_, more effectively reduced hemolysis and enhanced antimicrobial activity than did reference lipopeptides with similar hydrophobicity (C14-RR-NH_2_) and identical number of carbon atoms in the fatty acid chain (C16-RR-NH_2_).

## 2. Results and Discussion

### 2.1. Selection of a Position to be Substituted

Peptide hydrophobicity was determined by RP-HPLC analysis. To learn which amino acid position of a lipopeptide will result in the most significant change in retention time, two series of lipopeptides were synthesized–with decanoic (C10; peptides–**24**, **106**, **108**) and dodecanoic acid (C12; peptides–**25**, **107**, **109**) and a phenylalanine residue (Table 1). The change in the retention time (∆tR) is a difference between each lipopeptide and C10-RR-NH_2_ (tR’ = 24.30 min) or C12-RR-NH_2_ (tR’ = 32.58 min).

Within both peptide series (with C10 and C12) for peptides with phenylalanine residue at *N*-terminal position the most distinct change in retention time was observed. In general, when phenylalanine residue was closer to the *N*-terminus (fatty acid) then peptide hydrophobicity was higher. Presumably, interactions with stationary phase (C18 alkyl chain of column) are more effective when the hydrophobic region remains unperturbed by any non-hydrophobic residue to result in a longer retention time. Moreover, substitution in this position seems to be optimum to retain two well-defined regions of the lipopeptide molecule, hydrophobic and hydrophilic. This general observation is consistent with hydrophobicity coefficients determined by Tripet et al. [24] where hydrophobicity of phenylalanine (and other hydrophobic amino acid residues) increased in the order: intrinsic position (center, internal residue), *N*-terminal position, *N*-terminal residue with *N*^α^-acetyl. In other words, side chain hydrophobicity of amino acid residue among those three groups is highest when *N*-terminal position is substituted and acetyl group is attached to *N*-terminal amino group. Furthermore, results obtained by Domalaon et al. [25] show that disturbance of the hydrophobic domain can contribute to a significant decrease in antimicrobial activity of USCLs. Therefore, lipopeptides were synthesized with substituted *N*-terminal position (first amino acid residue; Fatty acid-XRR-NH_2_).

### 2.2. Hydrophobicity and Antimicrobial Activity of Reference Lipopeptides

**Figure 2:**
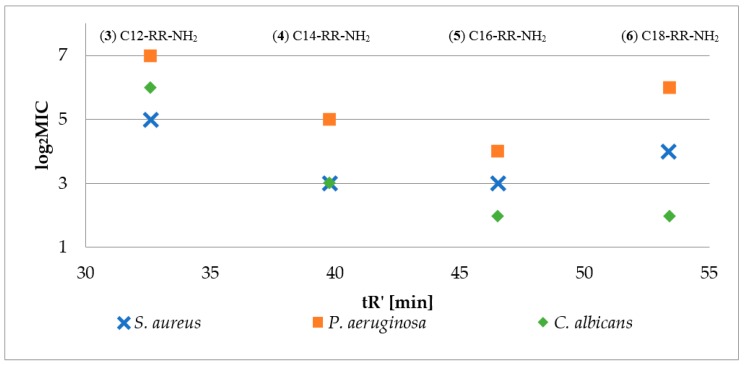
Antimicrobial activity (log_2_MIC) of reference lipopeptides (**3**–**6**) against *S. aureus ATCC 25923*, *P. aeruginosa ATCC 9027*, and *C. albicans ATCC 10231* and fatty acid chain length (nC) versus adjusted retention time.

Firstly, antimicrobial activity of reference lipopeptides (Cx-RR-NH_2_; Cx–fatty acid residue, x = {8, 10, 12, 14, 16, 18}) was determined against *S. aureus*, *P. aeruginosa* and *C. albicans*, to estimate optimum length of the fatty acid chain. To determine peptide hydrophobicity parameter (adjusted retention time) the peptides were analyzed by RP-HPLC in triplicate to give standard deviation less than 0.05. Antimicrobial activity of reference lipopeptides (log_2_MIC) versus peptide hydrophobicity (t_R_’) is presented in Figure 2. Logarithm MIC was used for a better presentation of the data. Moreover, peptides C8-RR-NH_2_ (**1**) and C10-RR-NH_2_ (**2**) did not inhibit the growth of the tested strains over the whole concentration range (0.5–256 µg/mL).

The results confirmed that the peptide hydrophobicity depends on the length of fatty acid chain. In this group the optimum one was found for C16 (**5**) which in the case of reference lipopeptides resulted in the highest activity against all three strains. Consequently, peptides with C8-C14 fatty acids and additional amino acid residue (X) at *N*-terminus were synthesized (Fatty acid-XRR-NH_2,_ Figure 1). It seems that the enhanced hydrophobicity of peptides with palmitic acid residue (C16) is adverse since lipopeptides with stearic acid residues (C18) were usually less active against the tested strains. Moreover, the authors believe that synthesis of four series with different fatty acid length (C8, C10, C12, and C14) is sufficient to notice the effect of proposed concept (fatty acid shortening and simultaneous amino acid addition at *N*-terminus) on biological properties and hydrophobicity. This thesis was supported by optimum length of fatty acid chain required to effectively disrupt bacterial membrane. For instance, in the study conducted by Nasompag et al. it was determined for similar USCLs Cx-KYR-NH_2_ (net charge +2) and it was found for those with 12–14 carbon atoms [26]. Furthermore, in the research conducted by Malina and Shai, short lipopeptides (10–12 carbon atoms) appeared to be less hemolytic than those with longer fatty acid chains [21].

### 2.3. Hydrophobicity of Lipopeptide Series

**Figure 3:**
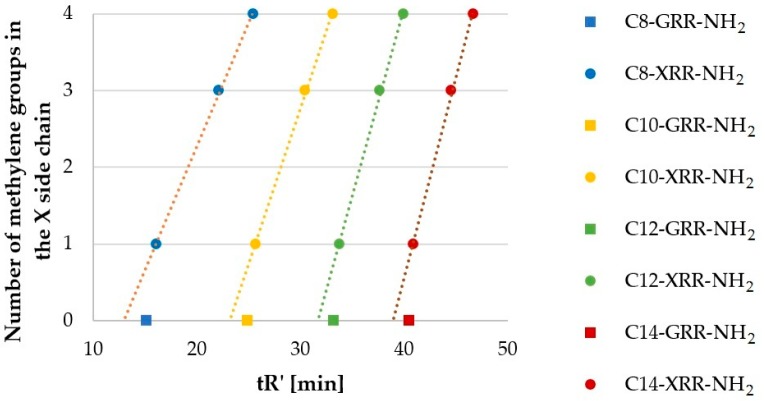
Hydrophobicity (tR’) of lipopeptides containing Gly (**27**–**30**), Ala (**7**–**10**), Nva (**67**–**70**), and Nle (**63**–**66**) versus number of methylene groups in the side chain.

Peptides with Gly (G, **27**–**30**), Ala (**7**–**10**), Nva (**67**–**70**), and Nle (**63**–**66**) formed another homologous series, which differed by methylene (-CH_2_-) moiety in the side chain. Results of RP-HPLC analyses (tR’) are presented in Figure 3. The ordinate refers to the number of methylene groups in the side chain of *N*-terminal (X) residues–0, 1, 3, and 4, respectively.

The number of carbon atoms in lipopeptide series (Figure 3) increased in the order Gly < Ala < Nva < Nle (0, 1, 3, 4). In each fatty acid series, lipopeptides with glycine residue (**27**–**30**) did not follow this trend. However, retention time of the remaining lipopeptides increased proportionally to the number of methylene groups in the side chain (R^2^ ranged between 0.9981 and 0.9994; USCLs with Gly were excluded). Interestingly, the impact of methylene moiety of the side chain and of the fatty acid chain on the lipopeptide hydrophobicity is different. For example, peptide C10-ARR-NH_2_ (**8**) was more hydrophobic than C8-NleRR-NH_2_ (**63**), despite the fact that the fatty acid chain of the latter is shorter by two methylene groups and its side chain is longer by three methylene groups. Presumably, this effect is due to side chain interactions with peptide back-bone and neighboring basic side chain of arginine. It is well documented that the charged moieties affect hydrophobicity of neighboring residues [27]. Moreover, the slope (Figure 3; the constant *a*; a_C8_ = 0.32, a_C10_ = 0.41, a_C12_ = 0.49, a_C14_ = 0.52) increases with the number of carbon atoms in the fatty acid chain. This finding is due to weakening impact of methylene group on hydrophobicity of the peptide with increasing molecular mass.

Another series of lipopeptides were those with aliphatic (X) side chain that differ in position of the methyl group in peptides with Ile (**35**–**38**), Leu (**43**–**46**) and Nle (**63**–**66**) residues. In general, peptides with methyl group that is closer to the backbone of lipopeptide were more hydrophilic. Retention time and the number of carbon atoms in the fatty acid chain (nC) of this series are presented in Table 2.

**Figure 4:**
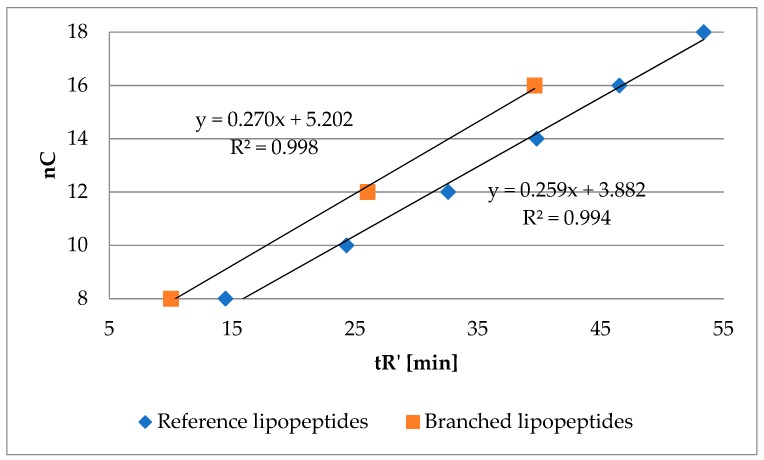
Number of carbon atoms in the fatty acid chain (nC) of branched lipopeptides (**103**–**105**) and reference lipopeptides (**1**–**6**) versus adjusted retention time.

Hypothetically, substitution of hydrogen atom in the hydrophobic residue (amino acid residue or branched fatty acid) with alkyl moiety might result in a more hydrophobic compound. However, with hydrophobicity lower than analogous lipopeptide with *N*-terminal straight fatty acid chain with the same number of carbon atoms as mentioned hydrophobic residue with added alkyl. In other words, hydrophobicity of methylene group measured by RP-HPLC might be different in the branched fatty acid and straight fatty acid chain. To verify this hypothesis, a series of lipopeptides with two arginine residues and branched fatty acids were analyzed, namely 2-ethylhexanoic (**103**), 2-butyloctanoic (**104**) and 2-hexyldecanoic (**105**) acids. Retention time of these compounds and of the reference lipopeptides (**1**–**6**) is presented in Figure 4. The number of carbon atoms in the fatty acid of branched lipopeptides is presented as a sum of carbon atoms (nC).

Branched lipopeptides with the same number of carbon atoms as reference ones were found to be more hydrophilic. These results are consistent with the previous statement where norleucine analogs (**63**–**66**) were the most hydrophobic in each series (Table 2).

Lipopeptides with Met (**47**–**50**), Met(O) (**51**–**54**), and Met(O2) (**55**–**58**) represent another series of compounds. However, this one differs in the number of oxygen atoms in the side chain (0, 1, and 2, respectively). In general, hydrophobicity of the lipopeptides increases as follows: Met(O) < Met(O2) < Met. Lipopeptides with Met(O) were the most hydrophilic due to the presence of S = O moiety which is partially ionized (S^+^-O^−^). With Met(O2), the ionization (O = S = O) is missing, but S = O bonds are polarized and the lone electron pairs of oxygen may participate in the hydrogen bond. For example, Lao et al. obtained similar results for amphipathic helical peptides containing oxidized methionine residues [28].

**Figure 5:**
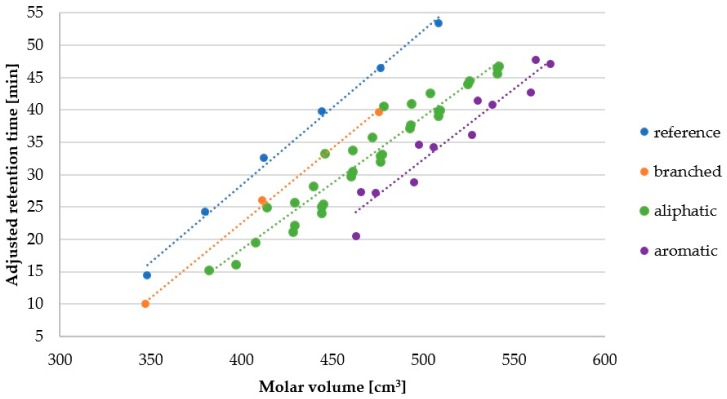
Molar volume of aliphatic (**7**–**10**, **27**–**30**, **35**–**38**, **43**–**46**, **63**–**74**, **91**–**94**), aromatic (**23**–**26**, **95**–**102**), branched (**103**–**105**) and reference (**1**–**6**) lipopeptides versus adjusted retention time.

Figure 5 presents the relation between retention time (hydrophobicity) and molecule size (see also Table S3). Molar volume (cm^3^) was calculated with ChemSketch 2012 freeware software (Advanced Chemistry Development, Inc., Toronto, ON, Canada, https://www.acdlabs.com/, 2019). The Figure 5 helps to answer the question–how the retention behavior is changing among groups of lipopeptides with similar molar volumes? 

In general, it seems that the groups can be ordered according to decreasing hydrophobicity as follows: reference, “branched” (branched fatty acid chain), aliphatic (Ala, Gly, Ile, Leu, Nle, Nva, Pro, Val), aromatic (Phe, Trp, Tyr). In fact, the difference between first two groups and two last groups is that additional peptide bond is polar and has a positive contribution to hydrophilicity. Dissimilarity between reference lipopeptides (**1**–**6**) and corresponding branched chain molecules (**103**–**105**) is (see also Figure 4) a reflection of a more compact structure and lower area that can effectively interact with stationary phase than that of linear chain [29]. Distinction between aliphatic (**7**–**10**, **27**–**30**, **35**–**38**, **43**–**46**, **63**–**74**, **91**–**94**) and aromatic (**23**–**26**, **95**–**102**) compounds relies on polarity. In aliphatic amino acid all carbon atoms are sp^3^ hybridized. But in aromatic amino acids some carbon atoms are sp^2^ hybridized. The difference in electronegativity of these two carbon atoms induce polarity of the side chain. Moreover, occurrence of heteroatoms such as nitrogen in tryptophan or p-hydroxyl group in tyrosine contribute to polarity of aromatic moiety. Another aspect is that chromatographic column in RP-HPLC can to some extent differentiate compounds with equal hydrophobicity but different shape (e.g., planar, non-planar) [30,31].

### 2.4. Peptide Hydrophobicity vs. Antimicrobial Activity and Hemolysis

In Table 3 the antimicrobial activity of the tested lipopeptides against reference strains is presented. Moreover, adjusted retention times (tR’) were considered. The hemolytic activity was determined for those lipopeptides with MIC values equal or lower than 32 µg/mL at least against one of the reference strains (SA–*Staphylococcus aureus* ATCC 25923, PA–*Pseudomonas aeruginosa* ATCC 9027, and CA–*Candida albicans* ATCC 10231). As a result, four reference lipopeptides (**3**–**6**) and their 45 analogs were selected for further studies. Hemolysis is presented as HC50 value–the concentration of compound that causes a 50% lysis of hRBCs. HC50 was calculated using an ic50.tk tool [32]. Full data can be found in the Supplementary Materials (Table S2).

Furthermore, for a better understanding of the relationship between antimicrobial activity and hydrophobicity, results are presented as log_2_MIC against adjusted retention time (lipophilicity). Relevant figures are attached as Supplementary Materials (Figures S1–S9). In general, antimicrobial activity of lipopeptides depended on their hydrophobicity, in agreement with the literature [33]. Regressions of log_2_MIC vs. tR’ for reference lipopeptides (**3**–**6**) and lipopeptides with aliphatic (**8**–**10**, **28**–**30**, **36**–**38**, **43**–**46**, **64**–**66**, **68**–**70**, **72**–**74**, **92**–**94**) and aromatic (**23**–**26**, **95**–**98**, **100**–**102**) amino acids were found to be either quadratic or linear (R^2^ between 0.9418 and 0.9985) with all three strains (Figures S1, S2, S4, S5, S7 and S8). Despite the fact that MIC depended on lipopeptide hydrophobicity, analogs with similar lipophilicity to reference lipopeptides had different MICs. Some lipopeptides with a branched fatty acid chain or additional amino acid residue have higher antimicrobial activity than the most promising reference lipopeptides including C10(6)-RR-NH_2_ (**105**), *N*-myristoylated (C14) lipopeptides with *N*-terminal (X) residue as F (**26**), I (**38**), L (**46**), M (**50**), Nle (**66**), Nva (**70**), R (**82**), V (**94**), W (**98**), Y (**102**), and *N*-lauroylated (C12) lipopeptides with F (**25**), Nle (**65**), and W (**97**). *N*-terminal amino acid residue in most of these compounds is hydrophobic. The only exception is arginine residue in C14-RRR-NH_2_ (**82**).

In general, in the case of all microorganisms lipopeptides represented the same pattern–higher net charge was associated with superior antimicrobial activity even if the lipopeptides had similar retention time. Simple linear regressions for lipopeptides with net charge +3 (analogs with H (**33**, **34**), K (**41**, **42**), R (**80**–**82**)–Figures S3, S6, and S9), lipopeptides with net charge +2 (Figures S1–S9; quadratic or linear regression) and those with net charge +1 (analogs with D (**17**, **18**), E (**21**, **22**)–Figures S3, S6, and S9), helped to visualize this rule. Despite the similar retention time of *N*-myristoylated analogs with net charge +3 there is an essential difference in hemolysis and selectivity. Hydrophobicity was measured in acidic conditions (mobile phase with 0.1% TFA) hence all basic residues were protonated, and in effect the analogs showed similar retention–C14-HRR-NH_2_ (**34**), C14-KRR-NH_2_ (**42**), C14-RRR-NH_2_ (**82**); 35.80, 35.66, and 36.76 min respectively. Determination of MIC values was performed in appropriate culture medium (CLSI guidelines) while determination of hemolysis was carried out in phosphate buffer saline, both at neutral pH (approx. 7.2–7.4). When considering pKa values of the side chains (Arg 12.10, Lys 10.67, His 6.04) [34] it is clear that histidine is not fully protonated under these conditions but only in a few percent (approx. 4%). In contrast to histidine, analogs with lysine and arginine are almost fully protonated. Moreover, the higher net charge seems to be beneficial for selectivity owing to lowering the % of hemolysis. Despite that C14-HRR-NH_2_ (**34**) has a relatively low hydrophobicity matching that of C14-KRR-NH_2_ (**42**) and C14-RRR-NH_2_ (**82**) it caused the highest hemolysis (HC50 of 50.31, 212.08, and 211.32 µg/mL respectively). In fact, it was even greater than that measured for the hydrophobic reference lipopeptide C14-RR-NH_2_ (**4**) (tR 39.79 min, HC50 68.43 µg/mL). For instance, Armas et al. noticed that arginine-rich USCLs with a higher net charge (additional arginine residues) suppressed hemolysis; whereas, antimicrobial activity against bacteria was comparable [9]. Moreover, Lohan et al. [35] have shown that USCLs with three basic residues had the highest antimicrobial activity among the tested lipopeptides (ornithine; net charge from +1 to +5); but the profile of hemolysis depended mainly on hydrophobicity and therefore on the length of the fatty acid chain. Under experimental conditions histidine imidazole can act as a hydrogen bond acceptor while guanidine moiety of arginine residue as a hydrogen bond donor. Furthermore, the side chain imidazole of histidine is aromatic ring and thus can interact with protonated arginine (guanidine moiety) and other organic and inorganic cations (cation-π interactions) [36,37]. Presumably, when the hydrogen bond acceptor is adjacent to arginine residue it can impede interactions of the protonated guanidine group with biological membranes and evoke negative effect on the antimicrobial activity and hemolysis. Other hydrogen bond acceptors are amide (asparagine, glutamine), hydroxyl (serine, threonine), methionine and its sulfoxide and sulfone, aspartic and glutamic acid, and Acm, but sulfur is a very poor hydrogen bond acceptor [38]. With aspartic and glutamic acid residues formation of salt bridges with arginine side-chain is plausible. Moreover, a lower net charge and occurrence of a negatively charged residue reduce interactions with the negatively charged bacterial membrane (electrostatic repulsions).

Interestingly, lipopeptides with aromatic amino acids (**23**–**26**, **95**–**98**, **100**–**102**) of hydrophobicity similar to that of those reference lipopeptides (**2**–**6**) showed a higher antimicrobial activity against *P. aeruginosa*. In other words, when the reference lipopeptide has equal MIC as an analog with aromatic amino acid then it is more hydrophobic (Figure S5). This can indicate some preference to aromatic amino acids. Improvement of biological activity of USCLs should not be based only on antimicrobial activity but also on cytotoxicity. In this study, the lytic activity of USCLs was evaluated against hRBCs. Generally, the rate of hemolysis caused by reference lipopeptides increased with elongation of the fatty acid chain, but a lipopeptide with octadecanoic acid (**6**) had a similar hemolysis to that of a lipopeptide with hexadecanoic acid (**5**). Interestingly, the lipopeptide with a branched fatty acid chain C10(6)-RR-NH_2_ (**105**) was characterized by a distinctly lower hemolysis than that of the reference lipopeptides with similar hydrophobicity (**4**) and the reference lipopeptide with identical number of carbon atoms (C16) in the fatty acid chain (**5**). In this study, HC50 of selected lipopeptides was determined to evaluate selectivity between pathogens (bacterial/fungal) membrane and human cell membrane. The analogs were compared to reference lipopeptides with the highest selectivity indexes (SI) and antimicrobial activity. Lipopeptide (**4**) (C14-RR-NH_2_) was selected when activity of analogs against *S. aureus* was considered. In case of *P. aeruginosa* and *C. albicans*, lipopeptide (**5**) (C16-RR-NH_2_) was selected as a reference compound. Antimicrobial activity of (**4**) and (**5**) was high and ranged between 4 and 16 µg/mL. These results are in agreement with the literature [5]. However, antifungal activity determined in this study was markedly higher (4 vs. 128 µg/mL) probably due to different culture media applied in antimicrobial susceptibility testing. In the present study, this was performed with RPMI-1640 medium with 2% d-glucose, while in the previous one the Sabouraud Dextrose Broth was used. It was demonstrated that culture media can affect the results of antimicrobial activity determination [39]. On the other hand, the antimicrobial activity against *S. aureus* (ATCC 25923 and clinical strains) and *E. coli* (ATCC 25922) of C16-RR-NH_2_ (**5**) and C16-KK-NH_2_ previously investigated by our team turned out to be similar [5,17]. Moreover, in this study MIC of C16-RR-NH_2_ (**5**) against *P. aeruginosa* (ATCC 9027) is equal to that of C16-KK-NH_2_ determined by Greber et al. [3]. Among the tested compounds, 18 lipopeptides were found to be more selective (SI) and simultaneously equally or more active than (**4**) or (**5**) against *S. aureus* (ATCC 25923) and *P. aeruginosa*; and only 8 lipopeptides against *C. albicans* (Table 3; gray shading).

### 2.5. Antimicrobial Activity of Selected Lipopeptides against Reference ESKAPE Strains

When MIC of USCL was equal or lower than 32 µg/mL at least against one of the reference strians (SA, PA or CA; Table 3) then it was selected for further studies with ESKAPE strains: E1–*E. faecium* ATCC 700221, S–*S. aureus* ATCC 33591, K–*K. pneumoniae* ATCC 700603, A–*A. baumannii* ATCC BAA-1605, P–*P. aeruginosa* ATCC 9027 (Table 3, Table S2), E2–*K. aerogenes* ATCC 13048.

To evaluate antimicrobial activity against ESKAPE strains and toxicity against hRBCs of the synthesized analogs, SI values were calculated. Unfortunately, none of the analogs were more or even equal active against *K. pneumoniae* and *K. aerogenes* as the most active reference lipopeptide (**6**). In spite of this, two analogs (**42**, **82**) were slightly more selective than the parent molecule. These results have shown that Gram-negative strains are less sensitive to cationic lipopeptides than are Gram-positive ones in agreement with previous studies [3,9]. This phenomenon is the outcome of the different structures of Gram-positive and Gram-negative bacterial cells. The latter have additional outer membrane composed mainly of lipopolysaccharide that can effectively prevent the passage of lipophilic molecules [40,41]. In general, drug resistance of Gram-negative bacteria can be explained in terms of efflux pumps (i.e., ArcAB) system that is responsible for effective pumping of antibiotics out of the cellular interior. Moreover, resistance towards cationic antimicrobial peptides can also be ascribed to proteolytic degradation, modification of bacterial surface and outer membrane [42,43,44]. In fact, *K. pneumoniae* produces a thick (0.56 ± 0.09 µm) polysaccharide capsule (antigen K) that can provide a resistance to several antimicrobials [45,46,47]. On the other hand, the antibiotic resistance of *K. aerogenes* results from the presence of modified porins, reduced outer membrane permeability, and also presence of efflux pumps [48,49]. With other ESKAPE strains, there are over a dozen (from 10 to 18) of more selective and equally or more active analogs than those of the parent molecules (Table 4; gray shading). Susceptibility to USCLs of both methicillin-resistant and methicillin-susceptible *S. aureus* strains, was usually comparable. There are eleven (**10**, **25**, **26**, **45**, **46**, **49**, **50**, **66**, **69**, **81**, **82**) USCLs being less active against MRSA strain and four ones (**54**, **74**, **97**, **98**) with lower MIC against this strain, while MIC was no more than two-fold different. Similarly, Joshi et al. [50] have not reported essential differences in antistaphylococcal activity of cationic lipopeptidomimetics against planktonic cultures of *S. aureus* ATCC 25923 and ATCC 33591. In that study, no significant differences in antimicrobial activity against *E. faecium* ATCC 29212 and vancomycin-resistant strain (ATCC 700221) were noticed.

## 3. Materials and Methods

### 3.1. Peptide Synthesis

Peptides were synthesized manually by solid-phase Fmoc/tBu methodology, where polystyrene resin modified by Rink Amide linker was used as solid support (Orpegen Peptide Chemicals GmbH, Heidelberg, Germany). Amino acids were purchased from Merck (Darmstadt, Germany)–Fmoc-L-Nle-OH, Fmoc-L-Nva-OH; and Orpegen Peptide Chemicals GmbH (Heidelberg, Germany)–Fmoc-L-Ala-OH, Fmoc-L-Asn(Trt)-OH, Fmoc-L-Arg(Pbf)-OH, Fmoc-L-Asp(OtBu)-OH, Fmoc-L-Cys(Acm)-OH, Fmoc-L-Gln(Trt)-OH, Fmoc-L-Glu(OtBu)-OH, Fmoc-Gly-OH, Fmoc-L-His(Trt)-OH, Fmoc-L-Ile-OH, Fmoc-L-Leu-OH, Fmoc-L-Lys(Boc)-OH, Fmoc-L-Met-OH, Fmoc-L-Phe-OH, Fmoc-L-Pro-OH, Fmoc-L-Ser(tBu)-OH, Fmoc-L-Thr(tBu)-OH, Fmoc-L-Trp(Boc)-OH, Fmoc-L-Tyr(tBu)-OH, and Fmoc-L-Val-OH. Fatty acids were acquired from Merck (Darmstadt, Germany)–octanoic acid (C8), decanoic acid (C10), dodecanoic acid (C12), tetradecanoic acid (C14), hexadecanoic acid (C16), octadecanoic acid (C18), 2-ethylhexanoic acid, 2-butyloctanoic acid, and 2-hexyldecanoic acid. Deprotection of the Fmoc group was performed with 20% (*v*/*v*) piperidine (Iris Biotech GmbH, Marktredwitz, Germany) solution in DMF (Honeywell, Seelze, Germany) for 15 min. Acylation was conducted with a mixture of DIC:OxymaPure:Fmoc-AA-OH (molar ratio 1:1:1) (DIC and OxymaPure; Iris Biotech GmbH, Marktredwitz, Germany) dissolved in DMF:DCM (1:1, *v*/*v*) in fourfold excess based on the resin for 1.5 h (DCM; Chempur, Piekary Slaskie, Poland). After deprotection and coupling reactions, resin was rinsed with DMF and DCM and subsequently the chloranil test was carried out. Peptides were cleaved from the resin using one of the mixtures; (A)–TFA, EDT, TIS, and water (92.5:2.5:2.5:2.5 *v*/*v*/*v*/*v*); (B)–TFA, TIS, phenol, and water (92.5:2.5:2.5:2.5, *v*/*v*/*v*/*v*); (C)–TFA, TIS, and water (95:2.5:2.5 *v*/*v*/*v*) (EDT, Merck, Darmstadt, Germany; TFA, Apollo Scientific, Denton, UK; TIS, Iris Biotech GmbH, Marktredwitz, Germany; Phenol, Merck, Darmstadt, Germany). Mixture (A) was used with peptides containing a methionine residue, mixture (B) was used with peptides containing a tryptophan or tyrosine residue, whereas mixture (C) for the remaining peptides. Cleavage from the resin was accomplished in TFA and scavengers mixture for 1.5 h with agitation. Afterwards peptides were precipitated with cool diethyl ether and lyophilized and purified by RP-HPLC. Pure fractions (>95%, by HPLC analysis) were collected and lyophilized. The identity of all compounds was confirmed by mass spectrometry (ESI–MS). MS spectra are attached as Supplementary Materials (Figure S1). Lipopeptides with Met(O) and Met(O2) residue were obtained by ozone oxidation of the parent molecule with methionine. Briefly, lipopeptide with Met residue was dissolved in 20% ACN/water (approx. 2 g/L; ACN, Merck, Darmstadt, Germany) and solution was bubbled with ozone (organic solvent partially reduces foaming). The sparger was immersed in peptide solution and ozone was produced by a generator. The generator and sparger were connected with silicone tube and gas flow was controlled by peristaltic pump (Kamush, LPeri 250, Lipopharm.pl, Zblewo, Poland). The progress of the reaction was monitored by RP-HPLC (Varian ProStar HPLC system, Varian, Inc., Palo Alto, CA, USA). This procedure lead to two main products with masses of +16 Da (Met(O)) and +32 Da (Met(O2)). After oxidation, peptides were purified and analyzed as described above (RP-HPLC, ESI-MS; Waters Alliance e2695 system with Waters 2998 PDA and Acquity QDa detectors; Waters, Milford, MA, USA). The calculated and measured *m*/*z* values are listed in the Supplementary Materials (Table S1).

### 3.2. Determination of Peptide Hydrophobicity with RP-HPLC

To determine peptide hydrophobicity, the Waters Alliance e2695 system with a Waters 2998 PDA Detector (software-Empower^®^3) was used. All analyses were carried out on a Phenomenex Luna C18(2) column (3.0 × 100 mm, 5 µm particle size, 100 Å pore size). The peptides were dissolved in water (0.1% TFA, *v*/*v*) to obtain a concentration of 1 g/L. UV detection at 214 nm was used, and aliquots (10 µL) were eluted with a linear 10–55% acetonitrile gradient in deionized water over 60 min at 25.0 ± 0.1 °C. The mobile phase flow rate was 0.5 mL/min. Both eluents contained 0.1% (*v*/*v*) of TFA. Each peptide sample was analyzed in triplicate.

### 3.3. Antimicrobial Activity

#### 3.3.1. Cultivation of Microorganisms

The *Acinetobacter baumannii* ATCC BAA-1605, *Candida albicans* ATCC 10231, *Enterococcus faecium* ATCC 700221, *Klebsiella aerogenes* ATCC 13048 (previously known as *Enterobacter aerogenes*), *Klebsiella pneumoniae* ATCC 700603, *Pseudomonas aeruginosa* ATCC 9027, *Staphylococcus aureus* ATCC 25923 and MRSA ATCC 33591, were acquired from the American Type Culture Collection (ATCC). Before running the tests, beads with cryo-protected microorganisms were transferred into fresh MHB (BioMaxima, Lublin, Poland) for bacteria or RPMI-1640 for fungi and incubated for 24 h at 37 °C. Then, the culture was seeded on the Mueller-Hinton agar (MHA) plates (BioMaxima, Lublin, Poland) for bacteria or Sabouraud dextrose agar (SDA) plates (BioMaxima, Lublin, Poland) for fungi and incubated as just mentioned. Cell densities for all assays were adjusted spectrophotometrically (Multiskan™ GO Microplate Spectrophotometer, Thermo Fisher Scientific, Vantaa, Finland) at 600 nm for bacteria and at 530 nm for fungi.

#### 3.3.2. Activity against Planktonic Cultures

The MICs were determined by broth microdilution method according to the Clinical and Laboratory Standard Institute guidelines [51,52]. For this purpose initial inoculums of bacteria (5 × 10^5^ CFU/mL) in MHB (BioMaxima, Lublin, Poland) and fungi (2 × 10^3^ CFU/mL) in RPMI-1640 with 2% d-glucose (Merck, Darmstadt, Germany) prepared with MOPS (pH 7.0 ± 0.1 at 25 °C; Merck, Darmstadt, Germany) were exposed to the ranging concentration of lipopeptides (0.5–256 µg/mL) and incubated at 37 °C for 18 h and 24 h respectively. The experiments were conducted on 96-well microtiter polystyrene plates (Kartell, Italy). The MIC was taken as the lowest peptide concentration at which a noticeable growth of microorganisms was inhibited. All experiments were conducted in triplicate.

### 3.4. Hemolysis Assay

The hemolysis assay was performed as described earlier according to the method of Avrahami and Shai [53,54]. Fresh human red blood cells (hRBCs) with anticoagulant (EDTA) were rinsed three times with a PBS (Merck, Darmstadt, Germany) by centrifugation at 800× *g* for 10 min and resuspended in PBS. Lipopeptides were serially diluted on 96-well microtiter polystyrene plate and hRBCs were added to reach final volume of 100 µL. Peptide concentration ranged between 0.5 and 256 µg/mL and final hRBCs concentration was 4% (*v*/*v*). Controls for zero hemolysis (blank) and 100% hemolysis consisted of hRBCs suspended in PBS and 1% of Triton-X 100 (Merck, Darmstadt, Germany), respectively. The plate was incubated for 1 h at 37 °C and then centrifuged (800× *g*, 10 min, 4 °C). Subsequently, supernatant was resuspended to new microtiter plates and absorbance at 540 nm were measured. All experiments were conducted in triplicate. Protocol of the study received approval from the local Bioethics Committee at the Medical University of Gdańsk (NKBBN/262/2019).

## 4. Conclusions

The present results give clear evidence that the shortening of fatty acid chain (C12 and C14) and simultaneous addition of amino acid residue at *N*-terminus leads to more selective and more active USCLs than compounds with relatively long straight fatty acid chain (C16 and C18). At the same time, the type of amino acid is crucial. Based on this study it is recommended to use F, Nle, W, and other hydrophobic amino acids. It seems that higher net charge (analogs with arginine and lysine) and branched fatty acid chain (2-hexyldecanoic acid) apparently improves USCLs selectivity. Presumably, this concept can successfully be applied to modify hydrophobic region of different types of antimicrobial lipopeptides i.e., linear, cyclic, double-chain, or antibiotic analogs. Hypothetically, modulation of the fatty acid chain length and type (branched fatty acid) of the adjacent amino acid may be also beneficial to other antimicrobial peptides where *N*-lipidation strategy has been used to improve biological properties; not only to USCLs [55,56]. Moreover, insertion of d-amino acids can be advantageous owing to enhanced protease stability. Change in configuration may result in different size of the peptide head-group and thus affect membrane fluidization [57,58]. Further studies should estimate toxicity of selected USCLs against different human cell lines and check the possibility of application of this idea to other lipopeptides, determine self-assembly characteristics, critical micellar concentration and peptide-membrane interactions, include quantitative structure–activity relationship (QSAR) studies, quantitative structure–toxicity relationship (QSTR), quantitative structure–retention relationship (QSRR) studies and molecular dynamics (MD) simulations to detect and identify interactions with bacterial, fungal and human cells membranes. 

## Figures and Tables

**Table 1:** Retention time of peptides **24**, **25**, **106**–**109**.

Code	Sequence	tR’ [min]	∆tR [min] *
24	C10-**F**RR-NH_2_	34.68	10.38
106	C10-R**F**R-NH_2_	31.44	7.14
108	C10-RR**F**-NH_2_	31.07	6.77
25	C12-**F**RR-NH_2_	41.45	8.87
107	C12-R**F**R-NH_2_	38.67	6.09
109	C12-RR**F**-NH_2_	38.02	5.44

* ∆tR was calculated by subtraction of tR’ of C10-RR-NH_2_ or C12-RR-NH_2_ from tR’ of the corresponding lipopeptide.

**Table 2:** Adjusted retention time of lipopeptides with Ile (**35**–**38**), Leu (**43**–**46**) and Nle (**63**–**66**) residues.

	Ile		Leu		Nle
Structure of the Side Chain	-CH_2_-CH(CH_3_)CH_2_CH_3_		-CH_2_CH_2_CH(CH_3_)CH_3_		-CH_2_CH_2_CH_2_CH_2_CH_3_
nC	Adjusted retention time [min]				
8	24.017	**<**	25.032	**<**	25.429
10	31.953		32.846		33.155
12	39.047		39.821		39.958
14	45.650		46.441		46.681

**Table 3:** Antimicrobial and hemolytic activities, selectivity indexes and retention time of the lipopeptides.

Code	Name	X	tR’ [min]	HC50 [µg/mL]	MIC SA [µg/mL] (SI)	MIC PA [µg/mL] (SI)	MIC CA [µg/mL] (SI)
1	C8-RR-NH_2_	Reference lipopeptides	14.46	-	>256	>256	>256
2	C10-RR-NH_2_		24.30	-	>256	>256	>256
3	C12-RR-NH_2_		32.58	>256	32 (>8)	128 (>2)	64 (>4)
4	C14-RR-NH_2_		39.79	68.43 (±1.41)	8 * (8.55) *	32 (2.14)	8 (8.55)
5	C16-RR-NH_2_		46.51	22.91 (±1.18)	8 (2.86)	16 * (1.43) *	4 (5.73)
6	C18-RR-NH_2_		53.39	25.19 (±0.67)	16 (1.57)	64 (0.39)	4 * (6.30) *
10	C14-ARR-NH_2_	A	40.92	41.08 (±0.62)	8 (5.14)	32 (1.28)	16 (2.57)
14	C14-C(Acm)RR-NH_2_	C(Acm)	43.24	39.72 (±1.25)	16 (2.48)	**16** **(2.48)**	8 (4.97)
18	C14-DRR-NH_2_	D	40.25	49.95 (±1.00)	64 (0.78)	128 (0.46)	32 (1.86)
22	C14-ERR-NH_2_	E	40.17	59.51 (±4.00)	64 (0.93)	128 (0.46)	32 (1.86)
24	C10-FRR-NH_2_	F	34.68	>256	**16** **(>16)**	**32** **(>8)**	64 (>4)
25	C12-FRR-NH_2_	F	41.45	91.21 (±16.38)	**4** **(22.80)**	**8** **(11.40)**	**8** **(11.40)**
26	C14-FRR-NH_2_	F	47.75	39.09 (±1.18)	**4** **(9.77)**	**8** **(4.89)**	**2** **(19.55)**
29	C12-GRR-NH_2_	G	33.20	>256	32 (>8)	**128** **(>2)**	64 (>4)
30	C14-GRR-NH_2_	G	40.53	31.94 (±1.99)	8 (3.99)	32 (1.00)	16 (2.00)
34	C14-HRR-NH_2_	H	35.80	50.31 (±0.52)	16 (3.14)	64 (0.79)	16 (3.14)
37	C12-IRR-NH_2_	I	39.05	120.40 (±1.55)	**8** **(15.05)**	**16** **(7.53)**	**16** **(7.53)**
38	C14-IRR-NH_2_	I	45.65	78.92 (±4.38)	**4** **(19.73)**	**4** **(19.73)**	**4** **(19.73)**
42	C14-KRR-NH_2_	K	35.66	212.08 (±6.12)	**8** **(26.51)**	**64** **(3.31)**	**32** **(6.63)**
45	C12-LRR-NH_2_	L	39.82	112.81 (±1.39)	**8** **(14.10)**	**16** **(7.05)**	**16** **(7.05)**
46	C14-LRR-NH_2_	L	46.44	29.50 (±1.05)	4 (7.38)	**4** **(7.38)**	**4** **(7.38)**
49	C12-MRR-NH_2_	M	37.95	206.50 (±9.14)	**8** **(25.81)**	**32** **(6.45)**	**32** **(6.45)**
50	C14-MRR-NH_2_	M	44.69	35.11 (±0.90)	**4** **(8.78)**	**8** **(4.39)**	**4** **(8.78)**
54	C14-M(O)RR-NH_2_	M(O)	39.18	116.28 (±1.77)	32 (3.63)	**64** **(1.82)**	32 (3.63)
58	C14-M(O2)RR-NH_2_	M(O2)	41.17	70.11 (±2.93)	16 (4.38)	**32** **(2.19)**	16 (4.38)
62	C14-NRR-NH_2_	N	38.71	64.37 (±2.23)	16 (4.02)	**32** **(2.01)**	16 (4.02)
64	C10-NleRR-NH_2_	Nle	33.16	>256	32 (>8)	**64** **(>4)**	64 (>4)
65	C12-NleRR-NH_2_	Nle	39.96	>256	**8** **(>32)**	**8** **(>32)**	**16** **(>16)**
66	C14-NleRR-NH_2_	Nle	46.68	37.54 (±1.39)	**4** **(9.39)**	**4** **(9.39)**	**4** **(9.39)**
69	C12-NvaRR-NH_2_	Nva	37.67	207.62 (±5.27)	**8** **(25.95)**	**32** **(6.49)**	**32** **(6.49)**
70	C14-NvaRR-NH_2_	Nva	44.53	42.04 (±1.38)	**4** **(10.51)**	**8** **(5.26)**	**4** **(10.51)**
73	C12-PRR-NH_2_	P	35.82	>256	32 (>8)	**64** **(>4)**	64 (>4)
74	C14-PRR-NH_2_	P	42.61	53.41 (±1.84)	16 (3.34)	**16** **(3.34)**	**8** **(6.68)**
78	C14-QRR-NH_2_	Q	38.52	72.31 (±3.00)	16 (4.52)	**32** **(2.26)**	32 (2.26)
81	C12-RRR-NH_2_	R	30.00	>256	**16** **(>16)**	**128** **(>2)**	128 (>2)
82	C14-RRR-NH_2_	R	36.76	211.32 (±9.57)	**4** **(52.83)**	**32** **(6.60)**	**16** **(13.21)**
86	C14-SRR-NH_2_	S	39.72	41.54 (±1.29)	16 (2.60)	32 (1.30)	32 (1.30)
89	C12-TRR-NH_2_	T	33.82	>256	32 (>8)	**128** **(>2)**	64 (>4)
90	C14-TRR-NH_2_	T	40.80	43.43 (±0.90)	8 (5.43)	32 (1.36)	8 (5.43)
93	C12-VRR-NH_2_	V	37.17	246.30 (±14.12)	**16** **(15.39)**	**32** **(7.70)**	**32** **(7.70)**
94	C14-VRR-NH_2_	V	44.02	30.73 (±0.41)	4 (7.68)	**8** **(3.84)**	8 (3.84)
96	C10-WRR-NH_2_	W	34.31	>256	**16** **(>16)**	**32** **(>8)**	**32** **(>8)**
97	C12-WRR-NH_2_	W	40.79	42.42 (±0.57)	**4** **(10.61)**	**8** **(5.30)**	8 (5.30)
98	C14-WRR-NH_2_	W	47.11	36.91 (±0.89)	**4** **(9.23)**	**8** **(4.61)**	**4** **(9.23)**
101	C12-YRR-NH_2_	Y	36.17	123.08 (±1.12)	**8** **(15.39)**	**16** **(7.69)**	32 (3.85)
102	C14-YRR-NH_2_	Y	42.73	46.52 (±5.24)	**4** **(11.63)**	**8** **(5.82)**	**4** **(11.63)**
105	C10(6)-RR-NH_2_	-	39.62	200.25 (±16.07)	**4** **(50.06)**	**8** **(25.03)**	**16** **(12.52)**
Number of more selective analogs than reference lipopeptide					22	34	20
Number of more selective and equal or more active analogs					18	18	8

Selectivity indexes (SI = HC50/MIC) and antimicrobial activity of lipopeptide analogs were compared to those of the most promising reference compounds marked with an asterisk (*). Moreover, selectivity indexes higher than those calculated for reference lipopeptides are bolded while gray shading was used for more selective analogs with equal or lower MIC values.

**Table 4:** Antimicrobial activity against ESKAPE strains, hemolysis, and selectivity index of selected lipopeptides.

Code	Name	X	HC50 [µg/mL]	MIC [µg/mL] (SI)				
				*E1*	*S*	*K*	*A*	*E2*
3	C12-RR-NH_2_	Reference compound	>256	32 (>8)	32 (>8)	>256 (-)	256 (>1.07)	>256 (-)
4	C14-RR-NH_2_		68.43 (±1.41)	8 * (8.55) *	8 * (8.55) *	256 (0.27)	64 (1.07)	256 (0.27)
5	C16-RR-NH_2_		22.91 (±1.18)	8 (2.86)	8 (2.86)	32 (0.72)	32 (0.72)	32 (0.72)
6	C18-RR-NH_2_		25.19 (±0.67)	8 (3.15)	16 (1.57)	16 * (1.57) *	32 * (0.79) *	16 * (1.57) *
10	C14-ARR-NH_2_	A	41.08 (±0.62)	8 (5.14)	16 (2.57)	256 (0.16)	**32** **(1.28)**	256 (0.16)
14	C14-C(Acm)RR-NH_2_	C(Acm)	39.72 (±1.25)	8 (4.97)	16 (2.48)	256 (0.16)	**16** **(2.48)**	256 (0.16)
24	C10-FRR-NH_2_	F	>256	32 (>8)	16 (>16)	>256 (-)	**64** **(>4)**	>256 (-)
25	C12-FRR-NH_2_	F	91.21 (±16.38)	**8** **(11.40)**	**8** **(11.40)**	256 (0.36)	**64** **(1.43)**	256 (0.36)
26	C14-FRR-NH_2_	F	39.09 (±1.18)	16 (2.44)	32 (1.22)	64 (0.61)	128 (0.31)	64 (0.61)
30	C14-GRR-NH_2_	G	31.94 (±1.99)	8 (3.99)	8 (3.99)	256 (0.12)	64 (0.50)	256 (0.12)
34	C14-HRR-NH_2_	H	50.31 (±0.52)	8 (6.29)	16 (3.14)	256 (0.20)	128 (0.39)	256 (0.20)
37	C12-IRR-NH_2_	I	120.4 (±1.55)	**8** **(15.05)**	**8** **(15.05)**	256 (0.47)	**64** **(1.88)**	256 (0.47)
38	C14-IRR-NH_2_	I	78.92 (±4.38)	**4** **(19.73)**	**4** **(19.73)**	64 (1.23)	**64** **(1.23)**	64 (1.23)
42	C14-KRR-NH_2_	K	212.08 (±6.12)	**8** **(26.51)**	**8** **(26.51)**	**128** **(1.66)**	**64** **(3.31)**	**64** **(3.31)**
45	C12-LRR-NH_2_	L	112.81 (±1.39)	16 (7.05)	16 (7.05)	256 (0.44)	**64** **(1.76)**	256 (0.44)
46	C14-LRR-NH_2_	L	29.50 (±1.05)	8 (3.69)	16 (1.84)	128 (0.23)	64 (0.46)	128 (0.23)
49	C12-MRR-NH_2_	M	206.5 (±9.14)	**16** **(12.91)**	**16** **(12.91)**	256 (0.81)	**64** **(3.23)**	256 (0.81)
50	C14-MRR-NH_2_	M	35.11 (±0.90)	8 (4.39)	8 (4.39)	128 (0.27)	**32** **(1.10)**	128 (0.27)
54	C14-M(O)RR-NH_2_	M(O)	116.28 (±1.77)	16 (7.27)	16 (7.27)	256 (0.45)	**64** **(1.82)**	>256 (-)
58	C14-M(O2)RR-NH_2_	M(O2)	70.11 (±2.93)	**8** **(8.76)**	16 (4.38)	256 (0.27)	**32** **(2.19)**	256 (0.27)
62	C14-NRR-NH_2_	N	64.37 (±2.23)	8 (8.05)	16 (4.02)	128 (0.50)	**32** **(2.01)**	128 (0.50)
65	C12-NleRR-NH_2_	Nle	>256	**8** **(>32)**	**8** **(>32)**	256 (>1)	**32** **(>8)**	256 (>1)
66	C14-NleRR-NH_2_	Nle	37.54 (±1.39)	8 (4.69)	8 (4.69)	32 (1.17)	64 (0.59)	32 (1.17)
69	C12-NvaRR-NH_2_	Nva	207.62 (±5.27)	**16** **(12.98)**	**16** **(12.98)**	256 (0.81)	**64** **(3.24)**	256 (0.81)
70	C14-NvaRR-NH_2_	Nva	42.04 (±1.38)	**4** **(10.51)**	**4** **(10.51)**	128 (0.33)	64 (0.66)	128 (0.33)
74	C14-PRR-NH_2_	P	53.41 (±1.84)	8 (6.68)	8 (6.68)	256 (0.21)	**64** **(0.83)**	256 (0.21)
78	C14-QRR-NH_2_	Q	72.31 (±3.00)	16 (4.52)	16 (4.52)	256 (0.28)	**64** **(1.13)**	256 (0.28)
81	C12-RRR-NH_2_	R	>256	**16** **(>16)**	32 (>8)	>256 (-)	**256** **(>1)**	>256 (-)
82	C14-RRR-NH_2_	R	211.32 (±9.57)	**8** **(26.42)**	**8** **(26.42)**	**128** **(1.65)**	**128** **(1.65)**	**128** **(1.65)**
86	C14-SRR-NH_2_	S	41.54 (±1.29)	16 (2.60)	16 (2.60)	128 (0.32)	**32** **(1.30)**	128 (0.32)
90	C14-TRR-NH_2_	T	43.43 (±0.90)	8 (5.43)	8 (5.43)	128 (0.34)	64 (0.68)	128 (0.34)
93	C12-VRR-NH_2_	V	246.3 (±14.12)	**16** **(15.39)**	**16** **(15.39)**	>256 (-)	**128** **(1.92)**	256 (0.96)
94	C14-VRR-NH_2_	V	30.73 (±0.41)	16 (1.92)	4 (7.68)	128 (0.24)	**32** **(0.96)**	128 (0.24)
96	C10-WRR-NH_2_	W	>256	**16** **(>16)**	**16** **(>16)**	>256 (-)	**64** **(>4)**	>256 (-)
97	C12-WRR-NH_2_	W	42.42 (±0.57)	8 (5.30)	**2** **(21.21)**	256 (0.17)	**32** **(1.33)**	256 (0.17)
98	C14-WRR-NH_2_	W	36.91 (±0.89)	8 (4.61)	**2** **(18.46)**	64 (0.58)	128 (0.29)	32 (1.15)
101	C12-YRR-NH_2_	Y	123.08 (±1.12)	16 (7.69)	**8** **(15.39)**	128 (0.96)	**32** **(3.85)**	128 (0.96)
102	C12-YRR-NH_2_	Y	46.52 (±5.24)	**4** **(11.63)**	**4** **(11.63)**	32 (1.45)	**32** **(1.45)**	64 (0.73)
105	C10(6)-RR-NH_2_	-	200.25 (±16.07)	**8** **(25.03)**	**4** **(50.06)**	>256 (-)	256 (0.78)	>256 (-)
Number of more selective compounds than reference lipopeptide				15	17	2	26	2
Number of more selective and equal or more active analogs				10	12	0	11	0

Selectivity indexes (SI = HC50/MIC) and antimicrobial activity of lipopeptide analogs were compared to the most promising reference compounds marked with an asterisk (*). Moreover, selectivity indexes higher than those calculated for reference lipopeptides are bolded while gray shading was used for more selective analogs with equal or lower MIC values.

## Supplementary Materials

The following are available online, Figure S1: The log_2_MIC of lipopeptides with aliphatic amino acid residue against *S. aureus* vs. tR’; Figure S2: The log_2_MIC of lipopeptides with aromatic amino acid residue against *S. aureus* vs. tR’; Figure S3: The log_2_MIC of lipopeptides with remaining amino residues against *S. aureus* vs. tR’; Figure S4: The log_2_MIC of lipopeptides with aliphatic amino acid residue against *P. aeruginosa* vs. tR’; Figure S5: The log_2_MIC of lipopeptides with aromatic amino acid residue against *P. aeruginosa* vs. tR’; Figure S6: The log_2_MIC of lipopeptides with remaining amino residues against *P. aeruginosa* vs. tR’; Figure S7: The log_2_MIC of lipopeptides with aliphatic amino acid residue against *C. albicans* vs. tR’; Figure S8: The log_2_MIC of lipopeptides with aromatic amino acid residue against *C. albicans* vs. tR’; Figure S9: The log_2_MIC of lipopeptides with remaining amino residues against *C. albicans* vs. tR’; Table S1: Peptides used in this study (ESI-MS); Table S2: Antimicrobial and hemolytic activities, selectivity indexes and retention time of lipopeptides (1–105); Table S3: Molecular volume of selected lipopeptides.

## Abbreviations

ACN	acetonitrile
Acm	S-acetamidomethyl group
ATCC	American Type Culture Collection
Boc	*tert*-butyloxycarbonyl group
CLSI	Clinical and Laboratory Standards Institute
DCM	dichloromethane
DIC	*N*,*N′*-diisopropylcarbodiimide
DMF	*N*,*N*-dimethylformamide
EDT	1,2-ethanedithiol
EDTA	ethylenediaminetetraacetic acid
ESI–MS	electrospray-ionization mass spectrometry
Fmoc	9-fluorenylmethoxycarbonyl group
HC50	lipopeptide concentration causing 50% hemolysis
hRBCs	human red blood cells
Met(O)/M(O)	methionine sulfoxide
Met(O2)/M(O2)	methionine sulfone
MIC	minimum inhibitory concentration
MOPS	3-(*N*-morpholino)propanesulfonic acid
Nle	norleucine
Nva	norvaline
Pbf	2,2,4,6,7-pentamethyl-dihydrobenzofuran-5-sulfonyl residue
PBS	phosphate-buffer saline
RP-HPLC	reverse-phase high-performance liquid chromatography
tBu	*tert*-butyl group
TFA	trifluoroacetic acid
TIS	triisopropylsilane
Trt	trityl group
USCLs	ultrashort cationic lipopeptides
