# Supplementary material for: Ultrashort Cationic Lipopeptides–Effect of N-Terminal Amino Acid and Fatty Acid Type on Antimicrobial Activity and Hemolysis
Source: Molecules. 2020 Jan 8;25(2):257. doi: 10.3390/molecules25020257 (PMC7024302; doi:10.3390/molecules25020257)
Supplement: Supplementary file 1 [file molecules-25-00257-s001.pdf]

Table S1. Peptides used in this study.

Fatty acid-XRR-NH_2

Code	Fatty acid	X	Average mass	Monoisotopic mass	Code	z	Measured m/z	Calculated m/z	
								Average	Monoisotopic
1	C8	-	455.598	455.333	1	1	456.41	456.61	455.33
2	C10	-	483.651	483.365		2	228.78	228.81	228.67
3	C12	-	511.704	511.396	2	1	484.48	484.66	484.37
4	C14	-	539.757	539.427		2	242.78	242.83	242.69
5	C16	-	567.810	567.458	3	1	512.47	512.71	512.40
6	C18	-	595.863	595.489		2	256.85	256.86	256.71
7	C8	A	526.676	526.370	4	1	540.52	540.77	540.43
8	C10	A	554.729	554.402		2	270.87	270.89	270.72
9	C12	A	582.782	582.433	5	1	568.57	568.82	568.47
10	C14	A	610.836	610.464		2	284.91	284.41	284.23
11	C8	C(Acm)	629.819	629.380	6	1	596.59	596.87	596.50
12	C10	C(Acm)	657.872	657.411		2	298.95	298.44	298.25
13	C12	C(Acm)	685.925	685.442	7	1	527.47	527.68	527.38
14	C14	C(Acm)	713.978	713.473		2	264.33	264.35	264.19
15	C8	D	570.685	570.360	8	1	555.53	555.74	555.41
16	C10	D	598.739	598.391		2	278.35	278.37	278.21
17	C12	D	626.792	626.423	9	1	583.53	583.79	583.44
18	C14	D	654.845	654.454		2	292.39	292.40	292.22
19	C8	E	584.712	584.376	10	1	611.61	611.84	611.47
20	C10	E	612.765	612.407		2	306.41	306.43	306.24
21	C12	E	640.818	640.438	11	1	630.52	630.83	630.39
22	C14	E	668.872	668.470		2	315.85	315.92	315.70
23	C8	F	602.772	602.402	12	1	658.52	658.88	658.42
24	C10	F	630.825	630.433		2	329.89	329.94	329.71
25	C12	F	658.878	658.464	13	1	686.56	686.93	686.45
26	C14	F	686.932	686.496		2	343.93	343.97	343.73
27	C8	G	512.649	512.355	14	1	714.6	714.99	714.48
28	C10	G	540.703	540.386		2	357.94	358.00	357.74
29	C12	G	568.756	568.417	15	1	571.49	571.69	571.37
30	C14	G	596.809	596.449		2	286.31	286.35	286.19
31	C8	H	592.737	592.392	16	1	599.52	599.75	599.40
32	C10	H	620.790	620.423		2	300.33	300.38	300.20
33	C12	H	648.844	648.455	17	1	627.55	627.80	627.43
34	C14	H	676.897	676.486		2	314.4	314.40	314.22
35	C8	I	568.756	568.417	18	1	655.58	655.85	655.46
36	C10	I	596.809	596.449		2	328.45	328.43	328.23
37	C12	I	624.862	624.480	19	1	585.51	585.72	585.38
38	C14	I	652.915	652.511		2	293.37	293.36	293.20
39	C8	K	583.770	583.428	20	1	613.53	613.77	613.41
40	C10	K	611.823	611.459		2	307.36	307.39	307.21
41	C12	K	639.877	639.491	21	1	641.57	641.83	641.45
42	C14	K	667.930	667.522		2	321.41	321.42	321.23

43	C8	L	568.756	568.417	22	1	669.6	669.88	669.48
44	C10	L	596.809	596.449		2	335.41	335.44	335.24
45	C12	L	624.862	624.480	23	1	603.53	603.78	603.41
46	C14	L	652.915	652.511		2	302.37	302.39	302.21
47	C8	M	586.794	586.374	24	1	631.56	631.83	631.44
48	C10	M	614.847	614.405		2	316.43	316.42	316.22
49	C12	M	642.900	642.436	25	1	659.57	659.89	659.47
50	C14	M	670.953	670.468		2	330.43	330.45	330.24
51	C8	M(O)	602.794	602.369	26	1	687.62	687.94	687.50
52	C10	M(O)	630.847	630.400		2	344.45	344.47	344.26
53	C12	M(O)	658.900	658.431	27	1	513.47	513.66	513.36
54	C14	M(O)	686.953	686.463		2	257.32	257.33	257.19
55	C8	M(O2)	618.793	618.364	28	1	541.5	541.71	541.39
56	C10	M(O2)	646.846	646.395		2	271.33	271.36	271.20
57	C12	M(O2)	674.899	674.426	29	1	569.55	569.76	569.43
58	C14	M(O2)	702.952	702.457		2	285.36	285.39	285.22
59	C8	N	569.701	569.376	30	1	597.58	597.82	597.46
60	C10	N	597.754	597.407		2	299.4	299.41	299.23
61	C12	N	625.807	625.439	31	1	593.46	593.75	593.40
62	C14	N	653.860	653.470		2	297.34	297.38	297.20
63	C8	Nle	568.756	568.417		3	198.57	198.59	198.47
64	C10	Nle	596.809	596.449	32	1	621.53	621.80	621.43
65	C12	Nle	624.862	624.480		2	311.38	311.40	311.22
66	C14	Nle	652.915	652.511		3	207.97	207.94	207.82
67	C8	Nva	554.729	554.402	33	1	649.54	649.85	649.46
68	C10	Nva	582.782	582.433		2	325.45	325.43	325.24
69	C12	Nva	610.835	610.464		3	217.29	217.29	217.16
70	C14	Nva	638.888	638.496	34	1	677.56	677.90	677.49
71	C8	P	552.713	552.386		2	339.42	339.46	339.25
72	C10	P	580.766	580.417		3	226.63	226.64	226.50
73	C12	P	608.819	608.449	35	1	569.54	569.76	569.43
74	C14	P	636.873	636.480		2	285.37	285.39	285.22
75	C8	Q	583.727	583.392	36	1	597.58	597.82	597.46
76	C10	Q	611.780	611.423		2	299.41	299.41	299.23
77	C12	Q	639.834	639.454	37	1	625.6	625.87	625.49
78	C14	Q	667.887	667.486		2	313.42	313.44	313.25
79	C8	R	611.784	611.434	38	1	653.6	653.92	653.52
80	C10	R	639.837	639.466		2	327.49	327.47	327.26
81	C12	R	667.890	667.497	39	1	584.64	584.78	584.44
82	C14	R	695.943	695.528		2	293	292.89	292.72
83	C8	S	542.675	542.365		3	195.66	195.60	195.48
84	C10	S	570.728	570.397	40	1	612.74	612.83	612.47
85	C12	S	598.782	598.428		2	307.06	306.92	306.74
86	C14	S	626.835	626.459		3	205.21	204.95	204.83
87	C8	T	556.702	556.381	41	1	640.79	640.88	640.50
88	C10	T	584.755	584.412		2	320.92	320.95	320.75
89	C12	T	612.808	612.444		3	214.51	214.30	214.17

90	C14	T	640.861	640.475
91	C8	V	554.729	554.402
92	C10	V	582.782	582.433
93	C12	V	610.835	610.464
94	C14	V	638.888	638.496
95	C8	W	641.808	641.413
96	C10	W	669.861	669.444
97	C12	W	697.914	697.475
98	C14	W	725.967	725.506
99	C8	Y	618.771	618.397
100	C10	Y	646.824	646.428
101	C12	Y	674.878	674.459
102	C14	Y	702.931	702.490
103	C6(2)	-	455.598	455.333
104	C8(4)	-	511.704	511.396
105	C10(6)	-	567.810	567.458

**Code**	**Fatty acid**	**Peptide**	**Average mass**	**Monoisotopic mass**
106	C10	-RFR-NH_2	630.825	630.433
107	C12	-RFR-NH_2	658.878	658.464
108	C10	-RRF-NH_2	630.825	630.433
109	C12	-RRF-NH_2	658.878	658.464

42	1	668.77	668.94	668.53
	2	335.1	334.97	334.77
	3	223.81	223.65	223.52
43	1	569.54	569.76	569.43
	2	285.38	285.39	285.22
44	1	597.57	597.82	597.46
	2	299.42	299.41	299.23
45	1	625.6	625.87	625.49
	2	313.47	313.44	313.25
46	1	653.63	653.92	653.52
	2	327.47	327.47	327.26
47	1	587.49	587.80	587.38
	2	294.35	294.40	294.19
48	1	615.52	615.86	615.41
	2	308.39	308.43	308.21
49	1	643.55	643.91	643.44
	2	322.44	322.46	322.23
50	1	671.59	671.96	671.48
	2	336.48	336.48	336.24
51	1	603.57	603.80	603.38
	2	302.43	302.40	302.19
52	1	631.63	631.85	631.41
	2	316.49	316.43	316.21
53	1	659.65	659.91	659.44
	2	330.45	330.46	330.22
54	1	687.64	687.96	687.47
	2	344.53	344.48	344.24
55	1	619.62	619.80	619.37
	2	310.45	310.40	310.19
56	1	647.63	647.85	647.40
	2	324.49	324.43	324.21
57	1	675.61	675.91	675.43
	2	338.41	338.46	338.22
58	1	703.63	703.96	703.47
	2	352.5	352.48	352.24
59	1	570.51	570.71	570.38
	2	285.83	285.86	285.70
60	1	598.56	598.76	598.42
	2	299.88	299.88	299.71
61	1	626.57	626.81	626.45
	2	313.94	313.91	313.73
62	1	654.59	654.87	654.48
	2	327.92	327.94	327.74
63	1	569.55	569.76	569.43
	2	285.35	285.39	285.22
64	1	597.58	597.82	597.46
	2	299.44	299.41	299.23

65	1	625.59	625.87	625.49
	2	313.42	313.44	313.25
66	1	653.59	653.92	653.52
	2	327.48	327.47	327.26
67	1	555.53	555.74	555.41
	2	278.34	278.37	278.21
68	1	583.57	583.79	583.44
	2	292.35	292.40	292.22
69	1	611.6	611.84	611.47
	2	306.41	306.43	306.24
70	1	639.65	639.90	639.50
	2	320.46	320.45	320.26
71	1	553.52	553.72	553.39
	2	277.37	277.36	277.20
72	1	581.54	581.77	581.43
	2	291.38	291.39	291.22
73	1	609.59	609.83	609.46
	2	305.4	305.42	305.23
74	1	637.62	637.88	637.49
	2	319.42	319.44	319.25
75	1	584.53	584.74	584.40
	2	292.88	292.87	292.70
76	1	612.55	612.79	612.43
	2	306.87	306.90	306.72
77	1	640.58	640.84	640.46
	2	320.97	320.92	320.74
78	1	668.61	668.89	668.49
	2	334.99	334.95	334.75
79	1	612.56	612.79	612.44
	2	306.98	306.90	306.72
	3	205.36	204.94	204.82
80	1	640.71	640.84	640.47
	2	320.99	320.93	320.74
	3	214.54	214.29	214.16
81	1	668.79	668.90	668.50
	2	335.09	334.95	334.76
	3	223.78	223.64	223.51
82	1	696.78	696.95	696.54
	2	349.05	348.98	348.77
	3	233.07	232.99	232.85
83	1	543.47	543.68	543.37
	2	272.31	272.35	272.19
84	1	571.55	571.74	571.40
	2	286.35	286.37	286.21
85	1	599.55	599.79	599.44
	2	300.4	300.40	300.22
86	1	627.57	627.84	627.47

	2	314.4	314.43	314.24
87	1	557.53	557.71	557.39
	2	279.44	279.36	279.20
88	1	585.61	585.76	585.42
	2	293.47	293.39	293.21
89	1	613.66	613.82	613.45
	2	307.54	307.41	307.23
90	1	641.71	641.87	641.48
	2	321.5	321.44	321.25
91	1	555.54	555.74	555.41
	2	278.36	278.37	278.21
92	1	583.57	583.79	583.44
	2	292.38	292.40	292.22
93	1	611.61	611.84	611.47
	2	306.44	306.43	306.24
94	1	639.63	639.90	639.50
	2	320.4	320.45	320.26
95	1	642.54	642.82	642.42
	2	321.91	321.91	321.71
96	1	670.57	670.87	670.45
	2	335.93	335.94	335.73
97	1	698.62	698.92	698.48
	2	349.96	349.96	349.75
98	1	726.64	726.98	726.51
	2	364.04	363.99	363.76
99	1	619.52	619.78	619.40
	2	310.39	310.39	310.21
100	1	647.58	647.83	647.44
	2	324.42	324.42	324.22
101	1	675.58	675.89	675.47
	2	338.43	338.45	338.24
102	1	703.61	703.94	703.50
	2	352.45	352.47	352.25
103	1	456.38	456.61	455.33
	2	228.73	228.81	228.67
104	1	512.45	512.71	512.40
	2	256.80	256.86	256.71
105	1	568.56	568.82	568.47
	2	284.86	284.41	284.23
106	1	631.54	631.83	631.44
	2	316.40	316.42	316.22
107	1	659.54	659.89	659.47
	2	330.42	330.45	330.24
108	1	631.76	631.83	631.44
	2	316.52	316.42	316.22
109	1	659.77	659.89	659.47
	2	330.52	330.45	330.24

Peptide hydrophobicity *vs* antimicrobial activity

Antimicrobial activity against *Staphylococcus aureus* ATCC 25923

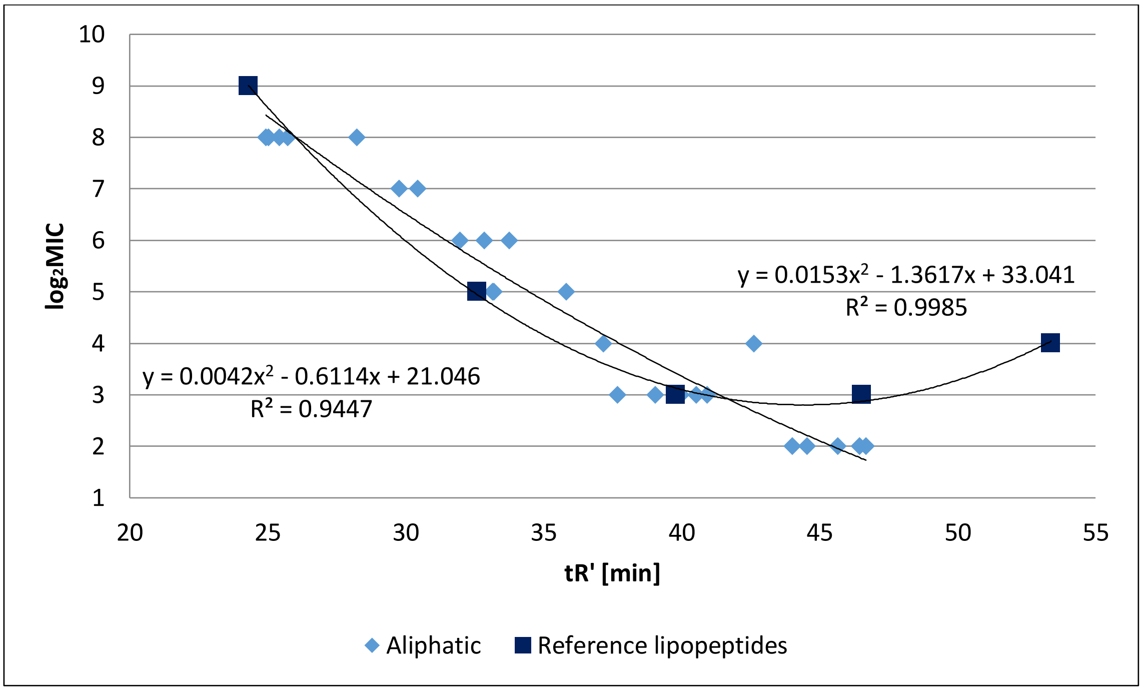

Figure S1. The $\log_2$MIC of lipopeptides with aliphatic amino acid residue against *S. aureus* *vs* tR'.

Aliphatic amino acid denotes Gly, Ala, Pro, Val, Leu, Ile, Nva, Nle.

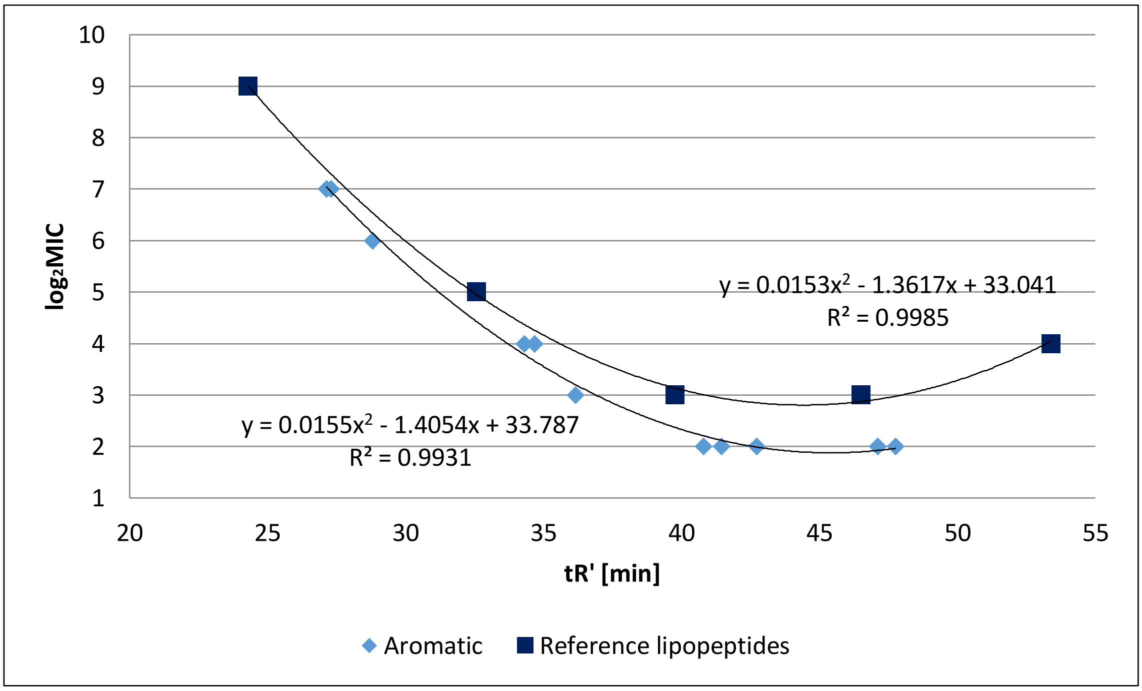

Figure S2. The $\log_2$MIC of lipopeptides with aromatic amino acid residue against *S. aureus* *vs* tR'.

Aromatic amino acid denotes Phe, Tyr, Trp.

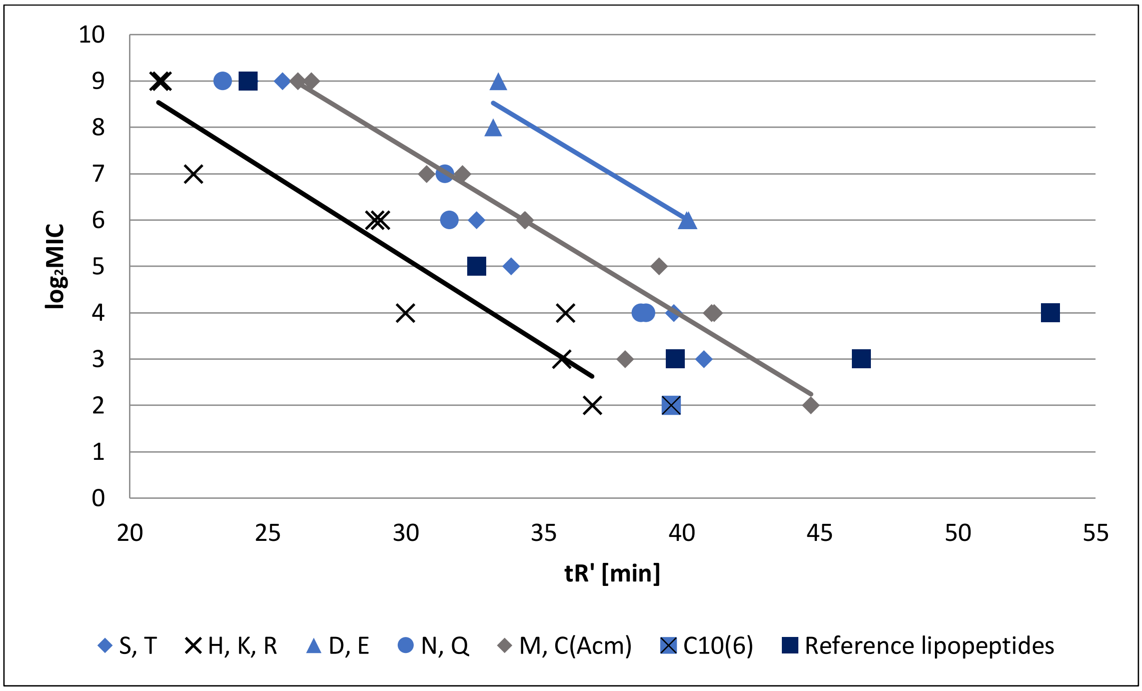

Figure S3. The $\log_2$MIC of lipopeptides with remaining amino residues against *S. aureus vs* tR'.

Remaining amino acid residues denotes Ser, Thr, His, Lys, Arg, Asp, Glu, Asn, Gln, Met, Met(O), Met(O2), C(Acm), moreover branched lipopeptide is included C10(6)-RR-NH_2.

Antimicrobial activity against *Pseudomonas aeruginosa* ATCC 9027

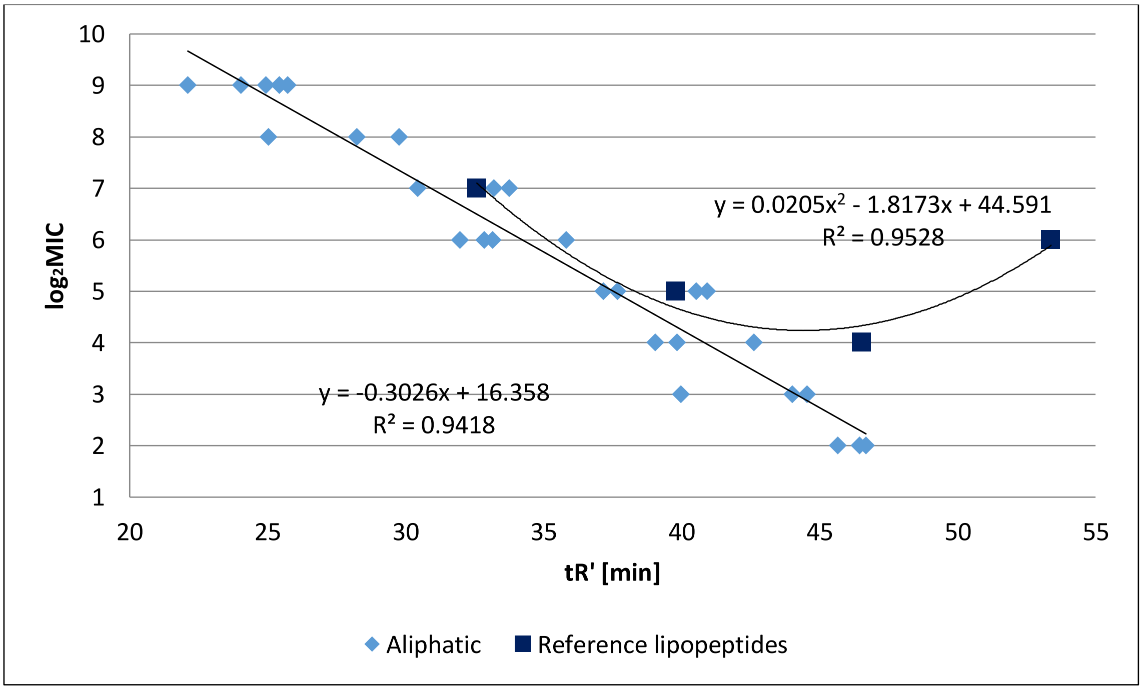

Figure S4. The $\log_2$MIC of lipopeptides with aliphatic amino acid residue against *P. aeruginosa vs* tR'.

Aliphatic amino acid denotes Gly, Ala, Pro, Val, Leu, Ile, Nva, Nle.

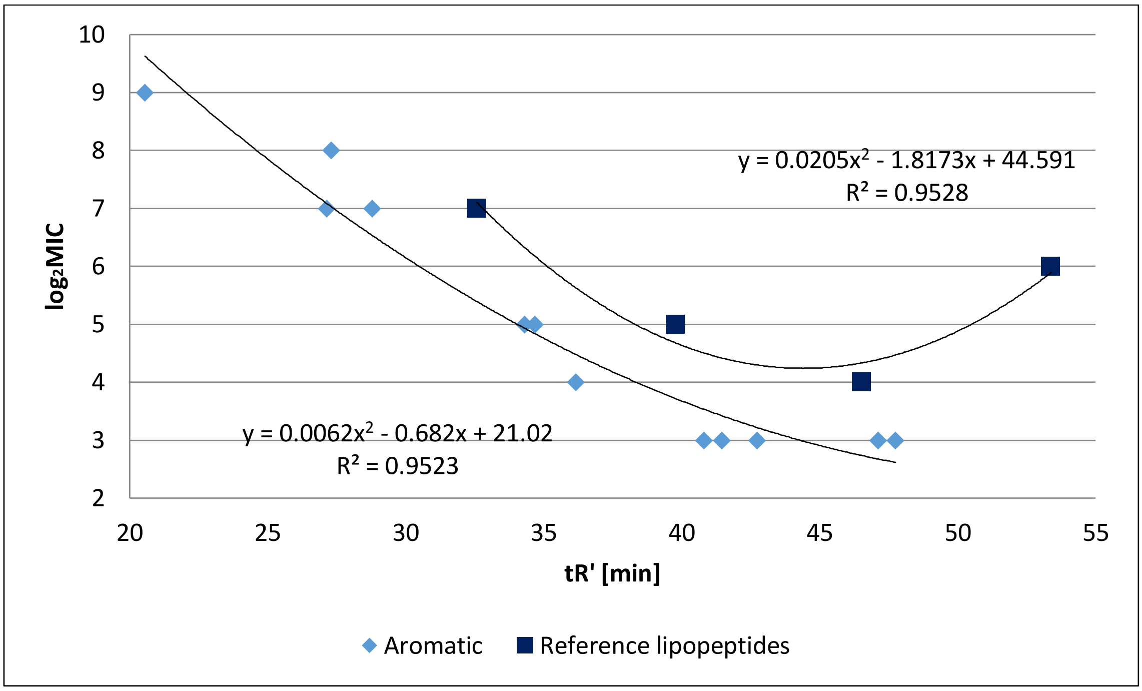

Figure S5. The log$_2$MIC of lipopeptides with aromatic amino acid residue against *P. aeruginosa vs* tR'.

Aromatic amino acid denotes Phe, Tyr, Trp.

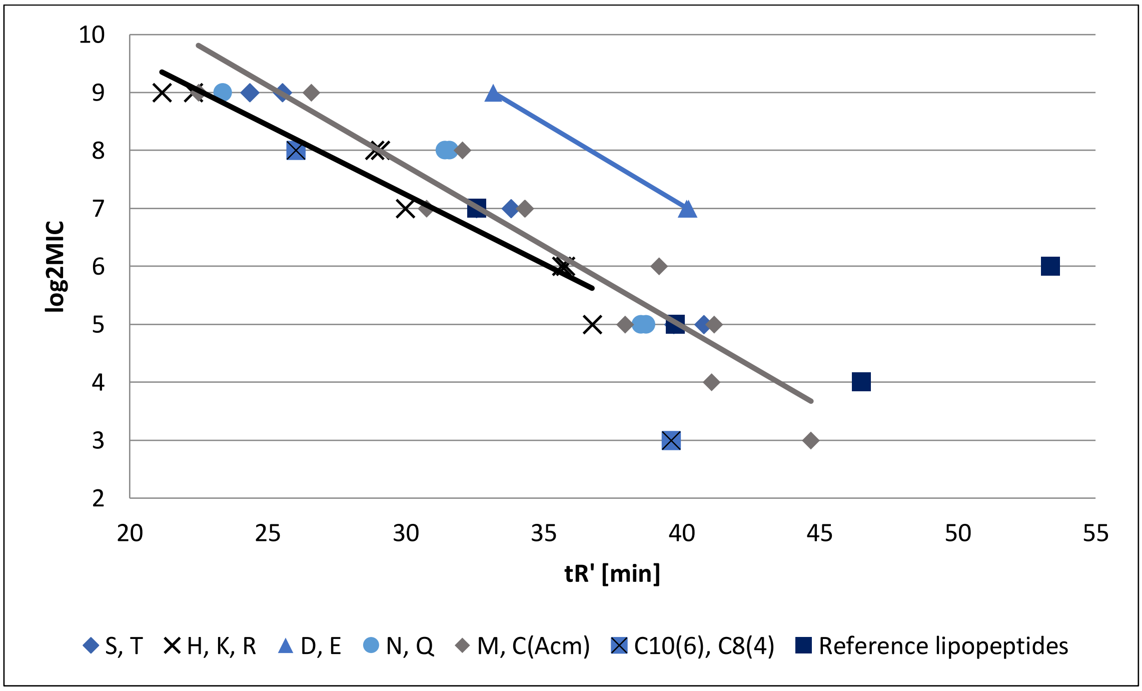

Figure S6. The log$_2$MIC of lipopeptides with remaining amino residues against *P. aeruginosa vs* tR'.

Remaining amino acid residues denotes Ser, Thr, His, Lys, Arg, Asp, Glu, Asn, Gln, Met, Met(O), Met(O2), C(Acm), moreover branched lipopeptides are included – C10(6)-RR-NH$_2$ and C8(4)-RR-NH$_2$.

Antimicrobial activity against *Candida albicans* ATCC 10231

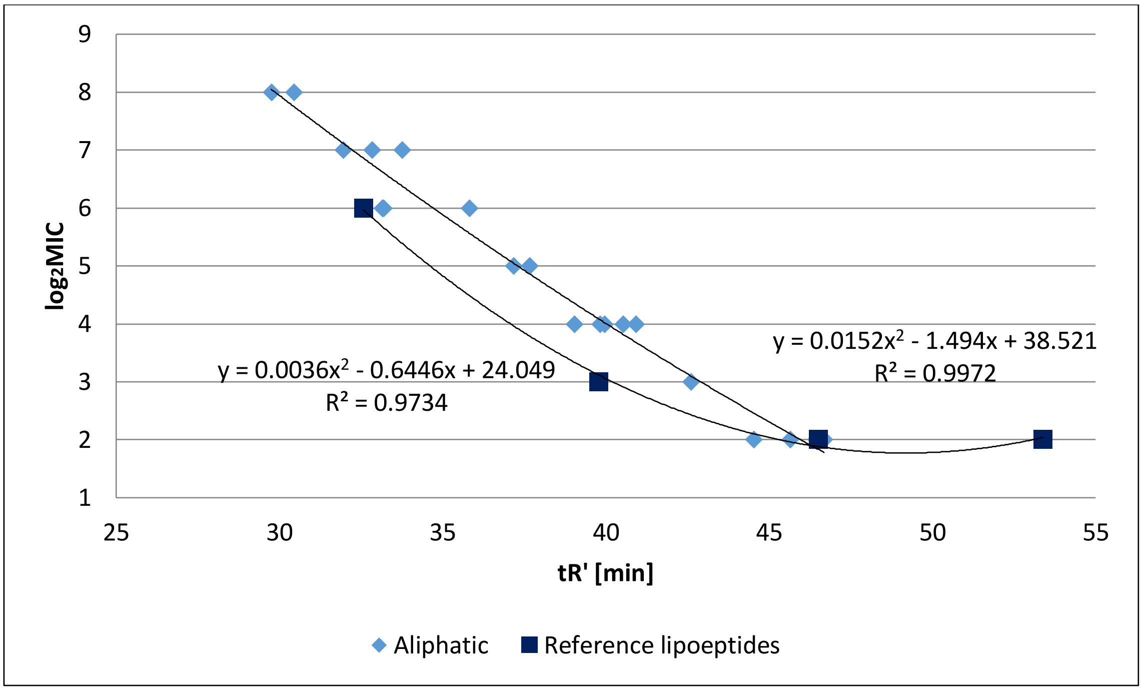

Figure S7. The $\log_2$MIC of lipopeptides with aliphatic amino acid residue against *C. albicans vs* tR'. Aliphatic amino acid denotes Gly, Ala, Pro, Val, Leu, Ile, Nva, Nle.

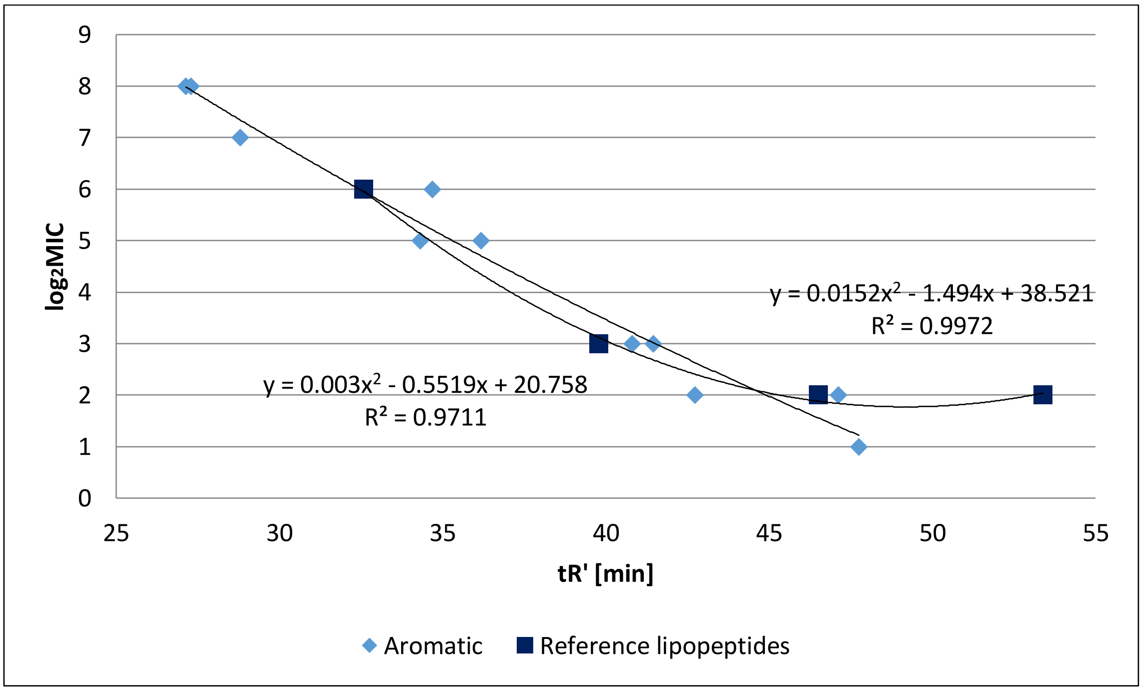

Figure S8. The $\log_2$MIC of lipopeptides with aromatic amino acid residue against *C. albicans vs* tR'. Aromatic amino acid denotes Phe, Tyr, Trp.

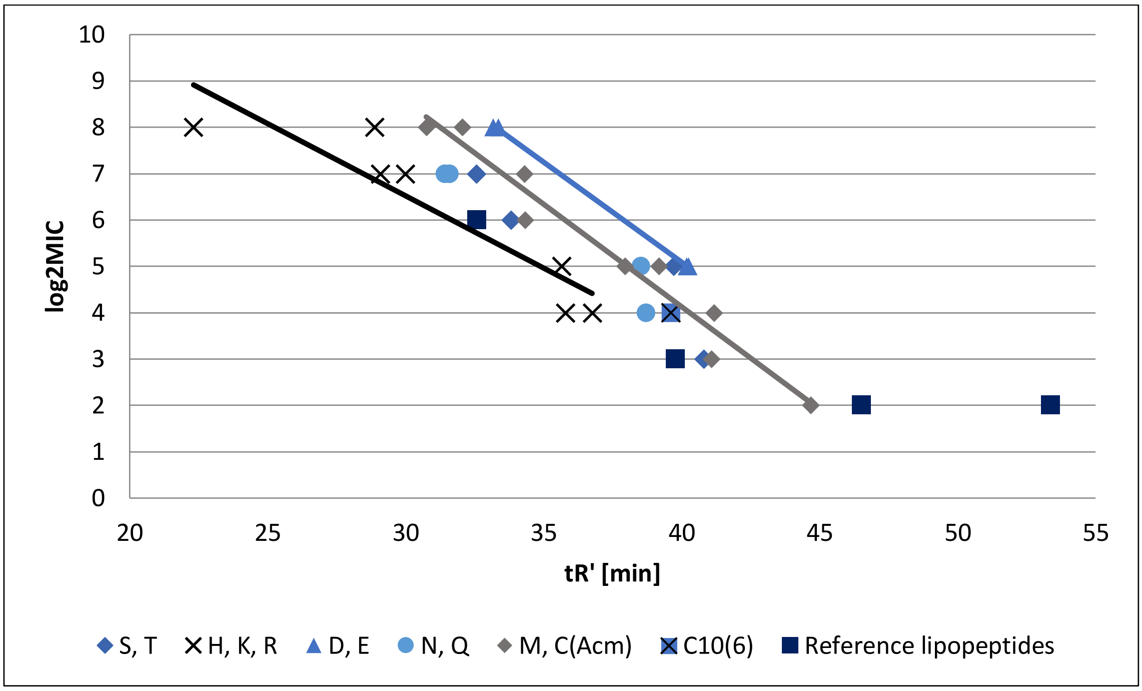

Figure S9. The log$_2$MIC of lipopeptides with remaining amino residues against *C. albicans vs* tR'.

Remaining amino acid residues denotes Ser, Thr, His, Lys, Arg, Asp, Glu, Asn, Gln, Met, Met(O), Met(O2), C(Acm), moreover branched lipopeptide is included – C10(6)-RR-NH$_2$.
